# Solution and high-pressure NMR studies of the structure, dynamics and stability of the cross-reactive allergenic cod parvalbumin Gad m 1

**DOI:** 10.1186/1939-4551-8-S1-A180

**Published:** 2015-04-08

**Authors:** Adolfo Moraes, Daniela Ackerbauer, Maria Kostadinova, Merima Bublin, Guilherme Augusto Oliveira, Fatima Ferreira, Fabio CL Almeida, Heimo Breiteneder, Ana Paula Valente

**Affiliations:** 1 Ufrj, Brazil; 2 Medical University of Vienna, Austria; 3 University of Salzburg, Austria; 4 Federal University of Rio De Janeiro, Brazil

## Background

IgE-mediated allergy to fish is a frequent cause of life-threatening allergic reactions. Allergen-specific immunotherapeutic approaches often make use of modified proteins that were designed to be hypoallergenic to reduce the risk of reactions. Since allergen-specific immunotherapy is the only treatment that provides long-term clinical benefits, determining the structure and molecular dynamics characteristics of allergens is necessary to understand how they interact with IgE and how to interfere with it by modifying the IgE-binding sites.

β-parvalbumins represent one of the largest animal food-allergen families and are considered cross-reactive pan-allergens in fish. The β-parvalbumin structure is characterized by the presence of three EF-hand motifs (helix-loop-helix) called AB, CD and EF, but only CD and EF can chelate calcium ions.

In order to contribute to the understanding of the allergenicity and the importance of the Ca2+-binding motifs for the stability of β-parvalbumins, we studied the major allergen of cod (*Gadus morhua*), Gad m 1, a member of the parvalbumin protein family

## Methods

The solution structure and the molecular dynamics of Gad m 1 were determined using NMR spectroscopy. Our strategy included high pressure to perturb the system and to evaluate crucial residues for structure stabilization at the atomic level. The Gad m 1-scFv complex structural characterization was done using chemical shift perturbation and the molecular dynamics of the complex was assessed by 15N-relaxation experiments.

## Results

Gad m 1 possesses the typical parvalbumin fold that is characterized by the presence of three domains, the two calcium-binding domains CD and EF, and the silent domain AB. High-pressure NMR revealed the important contribution of the AB domain to the protein fold stabilization. Although the Gad m 1 structure and accessibility of putative IgE epitopes are similar to parvalbumins from mackerel and carp, the charge of each of these sites is different.

## Conclusions

Our results offer new insights into the design of mutated proteins that would be stable in the non-allergenic apo form. Comparison of the Gad m 1 structure with other parvalbumins was done to understand the observed cross-reactivity.

